# A life-course approach to healthy ageing: maintaining physical capability

**DOI:** 10.1017/S0029665113003923

**Published:** 2014-01-23

**Authors:** Diana Kuh, Sathya Karunananthan, Howard Bergman, Rachel Cooper

**Affiliations:** 1 MRC University Unit for Lifelong Health and Ageing at UCL, 33 Bedford Place, London, UK; 2 Department of Epidemiology, Biostatistics and Occupational Health, McGill University, Montreal, QC, Canada; 3 Department of Family Medicine, McGill University, Montreal, QC, Canada

**Keywords:** Life course, Epidemiology, Healthy ageing, Physical capability, Cohort studies

## Abstract

Research on healthy ageing lacks an agreed conceptual framework and has not adequately taken into account the growing evidence that social and biological factors from early life onwards affect later health. We conceptualise healthy ageing within a life-course framework, separating healthy biological ageing (in terms of optimal physical and cognitive functioning, delaying the onset of chronic diseases, and extending length of life for as long as possible) from changes in psychological and social wellbeing. We summarise the findings of a review of healthy ageing indicators, focusing on objective measures of physical capability, such as tests of grip strength, walking speed, chair rises and standing balance, which aim to capture physical functioning at the individual level, assessing the capacity to undertake the physical tasks of daily living. There is robust evidence that higher scores on these measures are associated with lower rates of mortality, and more limited evidence of lower risk of morbidity, and of age-related patterns of change. Drawing on a research collaboration of UK cohort studies, we summarise what is known about the influences on physical capability in terms of lifetime socioeconomic position, body size and lifestyle, and underlying physiology and genetics; the evidence to date supports a broad set of factors already identified as risk factors for chronic diseases. We identify a need for larger longitudinal studies to investigate age-related change and ethnic diversity in these objective measures, the dynamic relationships between them, and how they relate to other component measures of healthy ageing. Robust evidence across cohort studies, using standardised measures within a clear conceptual framework, will benefit policy and practice to promote healthy ageing.

Abbreviations:
HALCyon: Healthy Ageing across the Life Course
NIH: National Institutes of Health
MRC: Medical Research Council

## Rationale for studying healthy ageing

Research on the factors that influence healthy ageing has become a priority of governments and funding agencies to inform strategies for reducing societal and individual costs of an ageing population. For example, living a long and healthy life is a key research priority theme in the current UK Medical Research Council (MRC) strategic plan^(^^1^^)^. Improvements in life expectancy that underlie population ageing should be heralded as a success but the societal costs of population ageing, particularly at a time of economic austerity, are more often the focus of debate. This is partly because there is little evidence, or inconsistent evidence, about whether improving trends in life expectancy are matched by similar trends in health gain, or the compression of morbidity. Mortality and morbidity trends do not necessarily change in parallel^(^^2^^)^. Whether population cohorts now reaching older ages are healthier than earlier born cohorts, and if they are healthier, what the main reasons for this are remain major areas of debate, and the answer is likely to be highly dependent on which aspect of health is being compared.

There are a number of conceptual and measurement challenges in the field of healthy ageing. One major challenge is the lack of an agreed conceptual framework, and no standard operational definition. A commonly cited review of ‘successful ageing’ notes twenty-nine different operational definitions across twenty-eight different studies up to 2005^(^^3^^)^. Since then there has been much more research in this area but little progression on the conceptual framework. The term ‘successful ageing’ is problematic: it encourages unattainable ideals of success and inappropriate ideas of failure; takes little account of the variation in environmental challenges that individuals face; appears to promote the idea that older people should act like younger people for as long as possible; and questions whether functional decline is inevitable, placing prime responsibility to delay decline on the individual. Measures used in studies of healthy ageing commonly use criteria that distinguish the least healthy individuals rather than identifying those in the best of health, and often do not investigate variability across the whole spectrum. For example, physical functioning, one of the most commonly included components in definitions of healthy ageing^(^^3^^)^, is often operationalised using self-reported measures of physical disability. Finally, we know little about whether the factors across life that promote healthy ageing are the mirror image of risk factors for the development of chronic disease, nor to what extent early life factors may be influential, as has been increasingly recognised for chronic disease risk. Systematic reviews and meta-analyses have become common in the chronic disease literature; a few exist for healthy ageing, limited in part by the lack of standard definitions of the outcome.

The first purpose of the present paper is to provide a life-course conceptual framework for healthy ageing, identifying the key components and inter-relationships that the research agenda needs to address. We then focus on one of these key components, physical capability, the capacity to undertake the physical tasks of daily living. The second purpose is to summarise our findings on physical capability measures from an ongoing MRC-funded review to produce guidelines on markers of healthy ageing. This review had two main objectives: to identify objectively assessed indicators of healthy ageing that are commonly used in population-based studies, easily applied in a range of settings, and exhibit meaningful variation from midlife onward; and to review the evidence for the suitability of these measures as markers of healthy ageing in terms of the patterns of age-related change in later life and prospective associations with important health outcomes. The third purpose of the present paper is to briefly summarise what is known about the lifetime determinants of physical capability, building on our recent reviews of the literature^(^^4^^)^. This includes the findings on physical capability from the Healthy Ageing across the Life Course (HALCyon) cross-cohort research programme, funded by the UK New Dynamics of Ageing cross council programme 2008–2013^(^^5^^)^. We conclude with a discussion of the most fruitful areas for further research on physical capability in order to be able to compare results across studies and build up a strong evidence base to inform policy and practice to promote healthy ageing.

## Conceptualising health, ageing and healthy ageing in a life-course framework

Health is a multi-dimensional concept, capturing how people feel, and how they function from the individual to the cellular level. Evolving classifications define diseases and disease risk using a constellation of signs, symptoms and assessments of impaired function. Health and disease reflect the ability of an organism to respond adaptively to environmental challenges^(^^6^^)^. Thus, a dynamic concept of health across life is needed, not just an assessment of health status at one point in time. Health can be seen as the ‘ability to adapt and self-manage’^(^^7^^)^, based on resilience to cope and maintain and restore one's integrity, equilibrium, and sense of wellbeing in three areas: biologically, in terms of physiological resilience; mentally, in terms of capacity to cope; and socially, in terms of the capacity to fulfil potential and obligations, manage independent living, and social participation.

Development and ageing refer to the changes in health with age. Health capital or ‘reserve’ is built up during development and reaches a peak or plateau at maturity. The progressive, generalised deterioration in function post maturity can be thought of as ‘biological ageing’ or ‘senescence’; the generally accepted disposable soma theory of ageing suggests that this is caused by the accumulation of molecular and cellular damage from environmental insults and chance^(^^8^^)^.

**Fig. 1.**
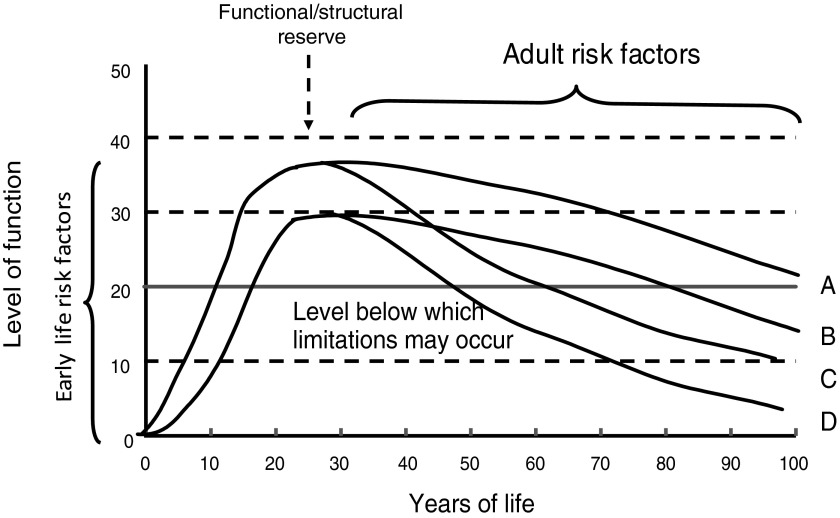
Life-course functional trajectories. A, normal development and decline; B, sub-optimal development resulting in reduced functional reserve at maturity; C, accelerated age-related decline; D, a combination of trajectories B and C.

A life-course perspective extends these ideas by investigating the biological and behavioural pathways that link physical and social exposures during gestation, childhood, adolescence and adult life, to changes in health and disease risk later in life^(^^4^^,^^9^^–^^11^^)^. Life-course functional trajectories (as illustrated in Fig. 1) for body functions (e.g. muscle, lung) or structures (e.g. bone mass), are used as a dynamic way of studying lifetime influences on health and disease risk; these trajectories capture the natural history of biological systems which display rapid growth and development during the prenatal, pre-pubertal, and pubertal periods, reaching a peak or plateau at maturity (‘structural or functional reserve’) and then a decline with age; there is much variation between individuals in the patterns and rate of decline that may be gradual or accelerated; and the age at onset of decline. Exposures in early life, particularly during a critical developmental window, may leave imprints on the structure or function of body systems; and epigenetic mechanisms may contribute to these processes. This developmental plasticity may affect reserve without appreciable effects on the rate of decline, or may interact with biological ageing processes to accelerate functional decline. Exposures after the developmental period can only affect the timing of onset and patterns and rate of decline.

### Defining healthy ageing

In our recent work^(^^4^^)^, we separated healthy ageing into healthy biological ageing and wellbeing. One essential component of healthy biological ageing was defined as the maintenance, post maturity, of optimal physical and cognitive functioning for as long as possible, delaying the onset and rate of functional decline. Other essential components are survival to old age, and delaying the onset of clinical disorders (i.e. health conditions where there is a consensus that medical treatment in terms of monitoring, and usually treatment, is required) and chronic diseases that accompany functional decline. In discussions of healthy ageing there is a tendency to forget that over 10% of individuals even from high income countries still do not survive to age 65 years and therefore do not have the chance to ‘age healthily’; rather the focus is on the declining proportion of the population in this group, down from about 40% at the beginning of the 20th century. Evidence from Europe and the USA^(^^12^^)^, suggests that overall the prevalence of chronic diseases is rising among older people due to some combination of the following factors that increase duration of time with disease: earlier diagnosis, decreased case fatality and diagnostic creep. Trends in functional limitations and disability are inconsistent across Organisation for Economic Co-operation and Development countries^(^^13^^)^, although overall there is some evidence that people are living longer without severe disability^(^^12^^)^. Recent comparisons of data on cohorts born in the first half of the 20th century is promising, showing, across cohorts, a decline in dementia in England and Wales^(^^14^^)^, and an improvement in cognitive functioning in Denmark^(^^15^^)^. As yet there is less evidence of cohort effects in later born cohorts, although cohort differences in obesity trends give cause for concern^(^^16^^)^.

Functional ageing may be assessed at the individual, body system or cellular levels. Sustaining performance for most physical tasks (such as rising from a chair or walking at normal speed) requires several body functions to operate. At the individual level we use the terms physical and cognitive capability to refer to the capacity to undertake the physical and mental tasks of daily living, to distinguish them from the physiological functions of the underlying body systems, and to focus equal attention on what individuals can, and cannot, do. At any of these levels, healthy biological ageing is about optimising function for as long as possible.

Wellbeing is distinct from healthy biological ageing, and evidence suggests that the lifetime trajectory of wellbeing is U-shaped, perhaps levelling off at the oldest ages, in contrast to the trajectory of development and ageing (Fig. 1). Wellbeing generally covers positive emotional health, and participation in valued social roles, engaging with others, leading meaningful lives, maintaining autonomy and independence.

Recently, we have proposed an integrated life-course model of ageing that suggests how these measures of healthy ageing inter-relate with each other and may be linked to physical, cognitive and emotional development in early life and to lifetime environmental factors and lifetime lifestyles^(^^9^^,^^11^^,^^17^^)^. This model elaborates our original life-course models^(^^18^^,^^19^^)^, taking more fully into account the heterogeneity in biological ageing, and incorporating two sources of resilience post maturity. The first is ‘compensatory reserve’, the ability of body systems to compensate physiologically or repair damage with varying degrees of success when faced with acute or chronic low-level challenges. The second source of resilience refers to the adaptations that individuals make to their behaviour or the environment, when faced with these challenges, in order to modify the effect on, or slow the rate of, functional decline.

## Physical capability measures as indicators of healthy biological ageing

Objective tests of physical capability with standardised assessment criteria have been developed since the 1980s; these measure capability along a continuum and have been incorporated into a growing number of population-based studies, particularly in the USA^(^^20^^,^^21^^)^. They include tests of grip strength, walking speed, chair rising and standing balance. These complement self-reports, improve validity and reproducibility, may more accurately capture change over time, and potentially reduce the influence of cognitive function, culture, language and education that can affect self-reports and make comparisons across studies problematic^(^^20^^)^. These tests also facilitate the study of variation across the full spectrum of function.

The focus of much of the research using these tests of physical capability, beyond their use as indicators of the current levels of functioning, is to provide evidence of their associations with subsequent morbidity, mortality and other health outcomes. A key aim is to identify thresholds or cut points that could be used in screening for those whose future health is likely to be compromised and who may require earlier intervention than others; however, most studies look at risk rather than prediction^(^^22^^)^. There is also a growing literature on the lifetime determinants of these tests of physical capability. In this research field, robust assessments of the evidence from systematic reviews and meta-analyses are still somewhat rare, but are increasingly being undertaken (see the ‘Lifetime determinants of physical capability’ section).

### Selection of physical capability measures

To select objective measures of physical capability for inclusion in the MRC review of markers of healthy ageing, we first identified all potentially relevant studies worldwide that had objectively assessed physical capability in adulthood and documented the measures they had each used. This was based on a review that one of the authors (R. C.) with a colleague undertook in 2008, as part of the HALCyon research collaboration that drew on sources including: a review paper on longitudinal studies of ageing^(^^23^^)^; relevant websites; and discussions with experts in the field of gerontology. We then identified other potentially relevant measures from the motor function domain of the National Institutes of Health (NIH) toolbox (http://www.nihtoolbox.org). For the latter, we consulted the NIH toolbox motor function team who, through a process of literature reviews, field surveys, and in-depth interviews with experts in the field, had identified five sub-domains that were considered critical for motor function^(^^24^^)^. This team had identified the most appropriate measure to objectively assess each of these subdomains across the full range of ages from 3 to 85 years and proposed a standardised method of assessment for each measure for inclusion in the toolbox.

We chose four of the five subdomains identified for use in the motor function domain of the NIH toolbox: locomotor function, strength, balance and dexterity. These accurately described the underlying functions that the most commonly used objective measures of physical capability assess. Locomotor function was assessed by tests of walking speed, timed ‘get up and go’, and chair rising. These measures also involve strength and balance and so assess aspects of multiple subdomains. Strength was assessed by tests of hand grip strength; standing balance by the one leg stand or tandem stands; and dexterity by the pegboard test. In the Supplementary material, we provide information on measurement protocols for these different tests, summarise common variations in protocols, equipment required, exclusion criteria commonly used and underlying body functions captured.

In summary, the Supplementary material provides evidence that all the tests of physical capability chosen are relatively quick, easy and cheap to perform with only grip strength and the pegboard test requiring special instruments; the other tests require some or all of the following: chair, tape measure, tape and stopwatch. There is also evidence that each of the tests is usually found to be valid and reliable. There was considerable variability between studies in the protocols of assessment^(^^25^^–^^30^^)^; however, attempts at standardisation across studies are now being made through initiatives such as the NIH toolbox.

**Fig. 2.**
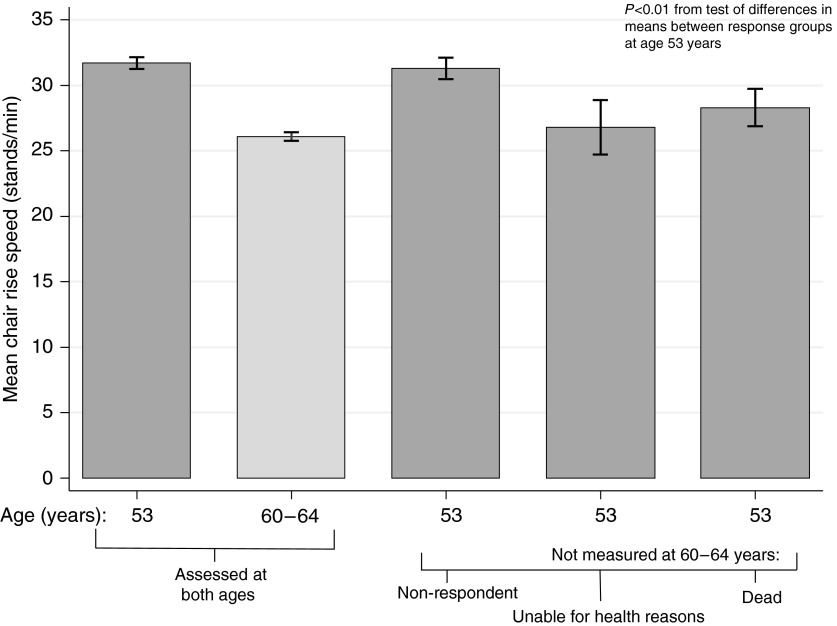
Mean chair rise speed (stands/min) at ages 53 and 60–64 years by response status in the Medical Research Council National Survey of Health and Development.

All the tests have exclusion criteria, and one challenge is how to handle the increasing proportion of people unable to perform these tests at older ages. Many studies simply exclude individuals without test results, but if data are missing because participants were unable to complete the test this is informative and, where possible, should be taken into account. For example, in the MRC National Survey of Health and Development, alternatively known as the 1946 British birth cohort study, grip strength, chair rise time and standing balance time have been measured at 53 years and again, where possible, at 60–64 years in 2500 study participants. It has been found that among those assessed at 53 years, those not assessed 10 years later because they had died in the intervening period, or were unable to do the tests for health reasons at 60–64 years, had significantly lower performance scores at 53 years than those measured at both ages (among whom a decline in mean levels of performance was observed). This is illustrated in Fig. 2 for chair rise speed. The mean level of performance at 53 years of other subsequent non-responders did not differ from the level of those with repeat data.

### Patterns of age-related change

As part of the MRC review of markers of healthy ageing, we assessed the evidence for age-related declines in mean levels of each of the selected measures of physical capability. As it was anticipated that we would not find systematic reviews examining this we aimed to identify individual studies of interest. Where possible we focused on studies with measures of longitudinal change but where insufficient studies with such data were available we also examined cross-sectional studies. The search terms used are provided in the Appendix; this search was supplemented by expert knowledge and web searches to identify relevant material, including unpublished work.

We found that the strength of the evidence varied widely between measures with the most evidence available for grip strength. Longitudinal data on grip strength across adult life show that mean levels of grip strength are at their peak in the fourth decade of life^(^^31^^–^^34^^)^, and then start to decline from the fifth or sixth decades^(^^31^^,^^33^^,^^34^^)^. Timing of the onset of decline in grip strength is similar for men and women^(^^26^^,^^31^^,^^33^^–^^40^^)^, but men start from a higher level and their rate of decline is faster^(^^33^^–^^35^^,^^41^^–^^44^^)^. Data on other physical capability measures is more limited and generally cross-sectional, which restricts inferences about age-related change but suggests a steady rate of age-related decline with men usually performing somewhat better than women at each age^(^^26^^–^^29^^,^^45^^–^^62^^)^.

### Associations with health outcomes

The final task of the MRC review of markers of healthy ageing was to assess the strength of the evidence for associations between our chosen physical capability measures and subsequent specified health outcomes, namely survival, disability, CVD, dementia, dependence and wellbeing, by systematic searches of the published literature to identify existing systematic reviews and meta-analyses. We focused on reviews of studies which had aimed to examine the specified associations longitudinally, excluding cross-sectional studies due to the possibility of reverse causality, and using community-dwelling samples, given likely confounding by morbidity in clinical/disease-specific samples. The Appendix provides the search terms used.

To date, two systematic reviews have shown an overall association between slower walking speed and all-cause mortality rates in older community dwelling populations, independent of age^(^^63^^,^^64^^)^. The first review also showed similar associations between grip strength, chair rise and standing balance performance and all-cause mortality rates^(^^63^^)^. Three other systematic reviews provide evidence that lower performance on these tests is also associated with greater risk of subsequent disability in terms of restrictions in activities of daily living^(^^29^^,^^65^^,^^66^^)^. A systematic review of the prospective associations of grip strength, walking speed, chair rise and standing balance performance with CVD, dementia and institutionalisation (as a marker of loss of independence) found some evidence that reduced physical capability was associated with higher risk of these health outcomes, but there were too few studies with comparable data for meta-analyses, thus limiting any definitive conclusions^(^^67^^)^. At the time of the literature search there were no systematic reviews of the prospective associations between measures of physical capability and wellbeing.

## Lifetime determinants of physical capability

The HALCyon research collaboration focused on the lifetime determinants of physical (and cognitive) capability and wellbeing. Here, we summarise the findings for the lifetime determinants of physical capability, drawing on a series of HALCyon publications, and the forthcoming HALCyon book on a life-course approach to healthy ageing^(^^4^^)^, in particular chapters 2, 10, 14 and 16. Investigating the lifetime determinants of physical capability is a continuing aim of what is now the HALCyon network, which brings together studies for cross-cohort comparisons, systematic reviews (according to the Preferred Reporting Items for Systematic Reviews and Meta-Analyses guidelines) and meta-analyses where appropriate, alongside in-depth studies from single cohorts. The focus of the original HALCyon research programme was on the relationships between physical capability and the following factors: lifetime socioeconomic position, body size and nutrition, and indicators of the underlying hypothalamic–pituitary–adrenal (axis), telomere length and genetic factors. To do this, measures of physical capability (grip strength, walking speed, chair rising and standing balance) from different HALCyon studies were harmonised^(^^26^^)^.

One HALCyon systematic review and meta-analysis showed modest associations between lower childhood socioeconomic position and slower walking and chair rise speeds that remained after adjustment for adult risk factors^(^^68^^)^; the mean difference found in walking speed translates into an 11% difference in mortality rates between those who were most and least deprived in childhood. Two in-depth studies in the National Survey of Health and Development, complemented this analysis. One showed that lifetime area level characteristics, as well as individual level characteristics, influence physical capability^(^^69^^)^. The second suggested that associations of childhood socioeconomic position with physical capability operate through neurodevelopmental and physical growth pathways^(^^70^^)^.

Another HALCyon systematic review and meta-analysis showed robust evidence that birth weight is positively related to subsequent grip strength; in children, young adults and in those at older ages^(^^71^^)^, suggesting that factors *in utero* or early postnatal life may leave long-term biological imprints on later life physical capability, in particular muscle. Studies from single cohorts show that growth in the pre-pubertal and pubertal periods is also important^(^^72^^–^^74^^)^.

There was cross-sectional evidence from eight of the HALCyon cohorts that greater adiposity was associated with worse physical performance^(^^75^^)^; the detrimental impact was greatest in the highest two-fifths of BMI and generally stronger in women than men. Weak grip strength was associated with poorer performance on tests of walking speed, chair rising and standing balance: again, associations were generally stronger in women than men; and particularly poor performance was seen in those in the lowest fifth of grip strength. BMI and grip strength were independently associated with these performance tests and had additive rather than interactive effects.

Across the HALCyon cohorts and other relevant studies, we showed no consistent evidence of associations between physical capability and common polymorphisms of: (1) *TERT*, a telomere maintenance gene^(^^76^^)^; (2) *ACTN3*, a genotype related to athletic status^(^^77^^)^; (3) genetic variants on the growth hormone and insulin-like growth factor-1 axis^(^^78^^)^. A HALCyon study has also shown that associations between change in telomere length and physical capability were weak and inconsistent^(^^79^^)^.

Somewhat more positive were the results of the relationships between markers of the hypothalamic–pituitary–adrenal axis and physical capability. Across four HALCyon cohorts and two other studies, cross-sectional data showed a larger diurnal drop in cortisol was associated with faster walking and chair rise speeds^(^^80^^)^; however, there was little evidence of associations with grip strength or standing balance. In a single-cohort study (Caerphilly prospective study), higher cortisol levels measured 20 years earlier, were associated with walking speed, in ways that suggested that the ability to mount a good stress-induced response may be a marker of a more reactive and healthier hypothalamic–pituitary–adrenal axis^(^^81^^,^^82^^)^.

HALCyon investigators also reviewed the literature on diet and physical capability and found that evidence from cross-cohort studies has been inconsistent and limited^(^^83^^)^, and not supported by trial evidence. There are major challenges of providing harmonised food or nutrient measures for cross-cohort studies. In addition, a recent literature review describing studies of the associations between nutritional factors and muscle strength^(^^84^^)^ highlighted that most observational studies to date have been cross-sectional, and existing longitudinal studies tend to have small sample sizes and relatively short follow-up. However, these studies do provide some evidence to suggest that diet quality and the intake of specific food groups and macronutrients are related to some measures of physical capability in later life^(^^85^^–^^88^^)^, although findings are not consistent. Furthermore, there is some evidence that these associations may operate across life; for example, evidence from the Boyd Orr study suggests that greater childhood milk intake was associated with better physical performance in old age, and there was some evidence that childhood intake of calcium, fat and protein were also important^(^^89^^)^.

There is more robust evidence from observational studies and randomised controlled trials of the importance of physical activity^(^^90^^)^.

## Suggestions for future research

While the extent of publications referenced attest to the considerable amounts of research undertaken on each of the physical capability measures, the variability between studies in the specific protocols employed often makes it difficult to compare and combine findings from different studies. For example, estimating the expected rate of decline in physical capability by age using data drawn from several sources is problematic. Very few studies have formally compared the different measures of physical capability in relation to a set of outcomes; so it is currently difficult to establish what the added value of assessing each additional measure is, and to recommend any order of priority. In a number of studies, a set of different objective tests of physical capability are often administered together^(^^91^^)^, such as the short physical performance battery^(^^92^^)^, and a total performance score is derived. Further work on the added value of each measure should establish whether the strategy of deriving an overall score of physical capability is of more prognostic value than considering each measure separately. The most appropriate approach is likely to depend on the specific research question being addressed.

We identified a number of studies that had examined age-related changes in population levels of objective measures of physical capability. However, there is a need for larger studies with longitudinal data in which these age-related patterns can be investigated further and variations in within-individual changes over time can also be characterised. This is likely to be especially important given that declines in mean levels of physical capability at the population level disguises the fact that not all people in a population are necessarily declining. Those people who are maintaining their physical capability despite their increasing age are likely to be an important group when studying healthy ageing.

Although several systematic reviews have examined associations of objective measures of physical capability with subsequent health outcomes, some measures have been studied more often than others. For example, grip strength and walking speed have been studied much more often than performance in the pegboard test, which has not been included in any of the reviews identified.

No reviews were identified that had examined the associations of physical capability with wellbeing, another component of healthy ageing. In light of this, we collected a common measure of wellbeing across five HALCyon cohorts, the Warwick Edinburgh Mental Wellbeing Scale. In a recent meta-analysis of the associations between physical capability and subsequent wellbeing in these cohorts^(^^93^^)^, we found that higher levels of physical capability were modestly associated with higher levels of subsequent mental wellbeing. Adjustment for age, gender, socioeconomic position, living alone, health status and neuroticism partially attenuated these associations. We have also found reasonable evidence that wellbeing may be protective for physical and cognitive capability, as well as being affected by functional decline^(^^94^^)^. However, this is still an area where further research is needed, as discussed elsewhere^(^^94^^)^.

Within HALCyon we investigated the associations of factors across life with physical capability using a number of well-established longitudinal studies in the UK, which have collected data at different life stages. As there is little or no ethnic diversity within the majority of HALCyon study populations, we were unable to explore the role of ethnicity, despite recognising its potential importance. From a national and international policy perspective it is going to be important in the future to explore differences by ethnicity and perform cross-national comparisons to identify which life-course associations are universal and which are contextual. This is especially important given emerging evidence from the UK, building on a larger body of work undertaken in the USA, that ethnic differences in physical capability and their determinants are clearly present in older populations^(^^95^^)^.

Another area for future research is to investigate the dynamic relationships between different measures of physical capability, between physical and cognitive capability, and between physical capability and the onset of clinical disorders or chronic diseases. Because of this gap in the literature, researchers from HALCyon and the Integrative Analysis of Longitudinal Studies on Aging network carried out a systematic review and meta-analyses of prospective associations between physical and cognitive capability^(^^96^^)^. Although many cross-sectional studies suggest that physical and cognitive capability are strongly correlated, we found far fewer longitudinal studies; indeed we identified only seven that had investigated ‘change’ in fluid cognition with ‘change’ in physical capability or lung function. Overall, findings were not sufficiently strong or consistent to support a common cause mechanism. Operationalisation and measurement challenges limited comparability, again identifying the need for common protocols and approaches.

**Fig. 3.**
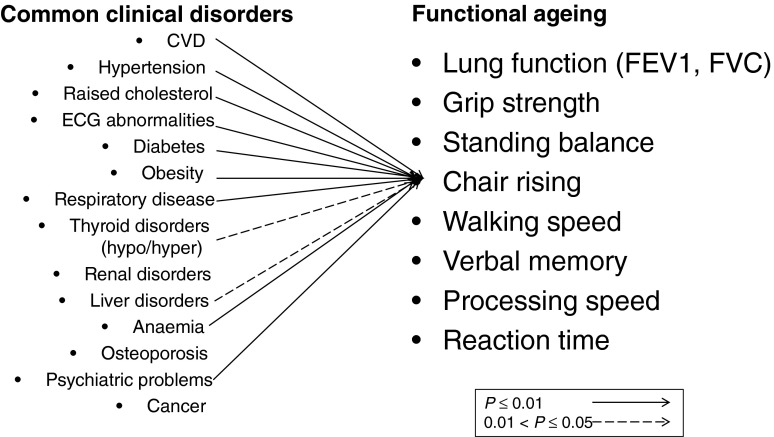
Patterns of association between common clinical disorders and chair rise performance at 60–64 years in the Medical Research Council National Survey of Health and Development. ECG, electrocardiogram; FEV1, forced expiratory volume in 1 second; FVC, forced vital capacity.

**Fig. 4.**
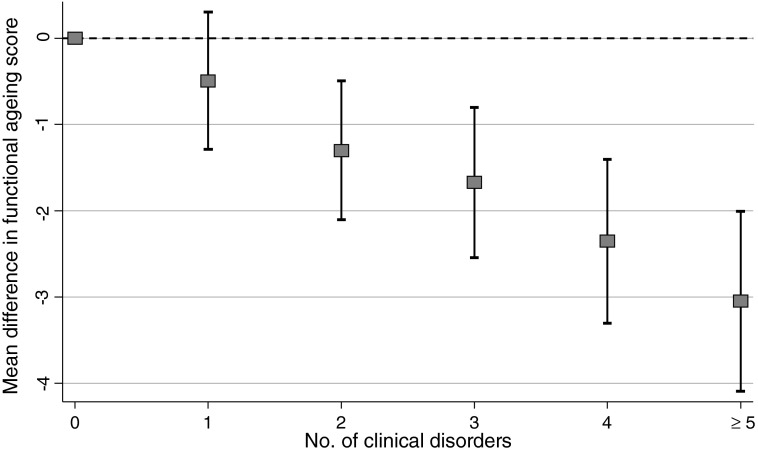
Differences in mean functional ageing score by number of clinical disorders in the Medical Research Council National Survey of Health and Development at age 60–64 years (sex adjusted).

In the National Survey of Health and Development, we plan to capture the unfolding relationships between physical and cognitive capability, and between capability and all the other components of healthy ageing, and the lifetime risk factors, mediators, and modifiers of these relationships. We recently showed that, on average, study participants had at least two out of fifteen common clinical disorders assessed at age 60–64 years, and less than one in six were disorder free^(^^97^^)^. Using cross-sectional data at 60–64 years, associations between each measure of physical capability and many of these fifteen disorders are observed; Fig. 3 illustrates this for chair rise performance. We have also recently published findings on the consistent associations in the National Survey of Health and Development between childhood socioeconomic circumstances and four measures of physical capability (walking speed, grip strength, chair rise and standing balance performance), two measures of lung function (forced expiratory volume 1 and forced vital capacity), three measures of cognitive capability (verbal memory, processing speed and reaction time) and an overall standardised measure of functional ageing at 60–64 years^(^^98^^)^. Fig. 4 shows a striking gradient between number of clinical disorders and this standardised composite measure of functional ageing.

In summary, what is required is longitudinal research that can compare findings on the different components of healthy ageing across studies and over time, and which can identify the key modifiable lifetime determinants to be targeted. The evidence to date on physical capability (and cognitive capability and wellbeing) is insufficient in these respects^(^^99^^)^, but so far largely supports the broad set of modifiable risk factors already identified for chronic diseases. Given the growing evidence that the majority of older people are ageing with chronic conditions, research on maximising physiological resilience, capacity to cope and maintaining social participation should become priorities. More robust evidence, using standardised measures within a clear conceptual framework, will benefit policy and practice to promote healthy ageing.

## Supplementary Material

Supplementary Material Supplementary information supplied by authors.

## Supplementary material

To view supplementary material for this article, please visit http://dx.doi.org/10.1017/S0029665113003923.

## Appendix: Search terms

*A. Search terms used for each physical capability measure*:
Walking speed and timed get up and go
(gait speed OR walking speed OR walk speed OR ‘get up and go’ OR TUG OR ‘timed get up and go’)
Chair rising
(chair ris* OR chair stand OR sit to stand)
Grip strength
(grip strength OR handgrip strength OR hand strength)
Balance (One leg stand and tandem stands)
(standing balance OR flamingo stand OR tandem stand OR postural control OR postural sway OR postural balance)
Pegboard test
(pegboard OR peg-board OR ‘peg test’ OR dexterity)
*B. Search terms used for patterns of age-related change*:
Physical capability measure (see terms in section A) AND (age difference OR trajectory OR age related change), limited to papers published since 1/1/1980
*C. Search teams used for associations with subsequent health outcomes*:
  1) Survival
    Physical capability measure (see terms in section A) AND (death OR mortality OR survival)
  2) Disability
    Physical capability measure (see terms in section A) AND disability
  3) Cardiovascular disease
    Physical capability measure (see terms in section A) AND (cardiovascular OR stroke OR CHD OR IHD)
  4) *Dementia* (restricted to clinically diagnosed outcomes)
    Physical capability measure (see terms in section A) AND (dementia OR Alzheimer's)
  5) Dependence
    Physical capability measure (see terms in section A) AND (dependency OR loss of independence OR dependence OR institutionalisation)
  6) Wellbeing
    Physical capability measure (see terms in section A) AND (wellbeing OR quality of life).
