# Supplementary material for: A life-course approach to healthy ageing: maintaining physical capability
Source: Proc Nutr Soc. 2014 Jan 23;73(2):237–48. doi: 10.1017/S0029665113003923 (PMC3981474; doi:10.1017/S0029665113003923)
Supplement: Supplementary Material — Supplementary information supplied by authors. [file S0029665113003923sup001.docx]

**Supplementary material**

**Details of methodological considerations for assessment of selected physical capability measures**

**a) Walking speed and timed get up and go test**

|  | **Walking speed** | **TImed get up and go (TUG) test** |
| --- | --- | --- |
| **Commonly used protocols** | Time taken to walk a specified short distance recorded and then usually converted to speed (in m/s).  Common variations in protocol:  *Starting position*: seated; standing stationary (static); moving (dynamic)  *Distance*: range of distances used including: 4m; 6m; 8 feet; 10m  *Pace*: normal; comfortable; as fast as possible; slow  *No. of trials*: 1, 2 or 3 (with most studies using only 1 trial and those with >1 trial using fastest or average speed) | Time taken to complete the following is recorded: from a seated start, the participant is instructed to stand up from the chair, walk 3m to a line (or other marker), cross the line, turn around, walk 3m back to chair and sit down again. Study participants are usually permitted to use a walking aid if required.  Common variations in protocol^(1)^:  *Chair:* Height of chair used varies between studies  *Pace:* Usually normal but in some studies participants have been instructed to walk quickly  *No. of trials:* 1 trial is usually conducted, but a practice trial is sometimes permitted  *Timing*: In some studies timing starts when the assessor says ‘go’ or ‘start’ and in other studies timing starts when participant stands up |
| **Protocol recommended by NIH toolbox** | Time taken to walk 4m from a standing start recorded. One practice and two timed trials undertaken (one at usual pace and one as ‘quickly as possible’) | Not included in toolbox |
| **Reports of validity and reliability** | See references^(2-5)^ | See references^(1,4,6-8)^ |
| **Equipment required** | Tape measure; tape (to mark out distance); stopwatch | Chair (with arms); tape measure and tape (to mark out 3m); stopwatch |
| **Exclusion criteria** | Unable to walk unaided (if people with walking aids are excluded)  Unable to stand | Inability to rise from a chair and walk without assistance from another person (although use of walking aids usually permitted)  Performance limiting problems (e.g. fear of falling) |
| **Other measurement considerations** | Need to ensure that there is enough space | Need to ensure that there is enough space |
| **Underlying functions** | LOCOMOTOR FUNCTION  Lower body strength and power; balance; energy; control of movement^(9)^ | LOCOMOTOR FUNCTION  Lower body strength and power; balance; coordination |
| **Other relevant information** | Reference values provided in studies by Bohannon and colleagues^(10,11)^  Impact of variations in protocol on measures recorded reviewed by Graham et al^(12)^ Influence on walking speed of allowing participants to use a walking aid investigated by Weiss et al^(13)^ | Test introduced by Podsiadlo and Richardson in 1991 as a modified version of a clinical measure of balance in older people and originally scored 1 to 5 based on observer’s assessment of participant’s likelihood of falling during the test^(6)^.  Reference values provided in a study of 527 older community-dwelling subjects by Pondal and del Ser^(14)^.  Study by Viccaro et al suggests that TUG may not add to information provided by walking speed when predicting health outcomes but may provide additional information for other purposes especially as it involves transitioning between different tasks^(15)^.  Influence of type of chair used on TUG performance in older people^(16)^. |

**b) Chair rising and grip strength**

|  | **Chair rising** | **Grip strength** |
| --- | --- | --- |
| **Commonly used protocols** | Participants start seated and then are timed standing up and sitting back down a specified number of times OR the number of times the participant is able to rise from a chair and sit back down again in a specified time is recorded.  Participants are usually encouraged to complete the test as fast as they can and are told to keep their arms folded.  Common variations in protocol:  *No. of rises performed:* If participants are asked to complete a specified number of rises they are usually asked to perform 5 or 10, although very old participants may be asked to demonstrate simply whether they can stand once from a chair unaided  *Timing*: In some studies timing starts when the assessor says ‘go’ or ‘start’ and in other studies timing starts when the participant stands up for the first time. In studies where a set number of rises are performed timing can be stopped when the participant stands for the last time or more commonly when the participant is fully seated back in the chair for the final time. | While seated participants are asked to squeeze the handle of a dynamometer as hard as they can (to elicit maximal performance) and the strength (i.e. muscle force) achieved is recorded (usually in kilograms, pounds or Newtons).  A review of protocols used to assess grip strength has been conducted by Roberts et al^(17)^  Common variations in protocol:  *No. of trials:* Often 2 or 3 attempts are undertaken per hand with the maximum or average value achieved used in analyses  *Hands used*: In some studies grip strength is measured in the dominant hand only but in many studies it is recorded in both hands  *Dynamometer:* A range of different hydraulic and electronic dynamometers are available to measure grip strength, the most commonly used is the Jamar dynamometer  *Position of the body:* Range of different positions for the wrist, forearm, elbow and shoulder reported, see Roberts et al^(17)^ |
| **Protocol recommended by NIH toolbox** | Not included in toolbox | Adapted from the protocol of the American Society of Handy Therapy. Participants sit in a chair with their feet on the ground and their elbow bent to 90 degrees, their arm against the trunk and wrist at neutral. They are then instructed to squeeze a dynamometer which they are given to hold in their hand (Jamar Plus Digital recommended) as hard as they can for a count of three. A practice trial is conducted followed by one recorded trial in each hand. |
| **Reports of validity and reliability** | Systematic review of reliability of the chair rise test (including 5 rises) reports that in most settings reliability is good to high^(18)^. | See references^(17,19-22)^ |
| **Equipment required** | Chair (without arms); stopwatch | Handgrip dynamometer; Chair with arms |
| **Exclusion criteria** | Inability to stand; Severe cardiorespiratory disease or untreated hypertension; Hip or knee replacement or other severe problems with hips or knees | Unable to use hands (e.g. due to hand osteoarthritis); recent surgery to the hands; swelling, inflammation, severe pain or recent injury to hands; high blood pressure |
| **Other measurement considerations** | There are differences in the number of chair rises it is appropriate to ask people of different ages to complete.  Floor effects may occur at older ages as an increasing percentage of participants find themselves unable to perform the test^(23)^ | Most dynamometers have different handle settings to allow for variation in the size of people’s hands.  If multiple dynamometers are used within a study these will need to cross-calibrated. Some form of calibration will also be required for cross-study comparisons. |
| **Underlying functions** | STRENGTH AND LOCOMOTOR FUNCTION  Lower body muscle power and strength; balance, coordination; cardiovascular and respiratory fitness (especially where test involves undertaking 10 rises) ^(24-27)^ | STRENGTH specifically isometric (upper body) muscle strength |
| **Other relevant information** | Findings from work undertaken while developing the NIH toolbox suggest that this test may not have sufficient discriminatory power at younger ages and because of this and the fact that the test does not measure only lower body strength it was not included in the NIH toolbox. However, it may still be a useful marker of healthy ageing.  Reference values for older populations reported in a study by Bohannon^(28)^. | It has been reported that grip strength and measures of lower body strength (such as knee extension strength) reflect a common underlying construct and that it is appropriate to characterise muscle strength, with caution, using a limited number of measures^(29-31)^. As a result we have chosen not to include knee extension strength in these guidelines. However, as the correlation between grip strength and knee extension strength decreases with increasing age, if costs and time allow for more than one test of strength to be included in a data collection, knee extension strength should also be considered.  Influence of allowing participants to select their own hand position reported by Boadella et al^(32)^. A number of comparisons of different dynamometers have been reported^(33-35)^.  Reference values reported in a number of studies^(35-37)^. |

**c) One leg stand, tandem stands and pegboard test**

|  | **Flamingo stand/ One leg stand (OLS)** | **Tandem stands** | **Pegboard test** |
| --- | --- | --- | --- |
| **Commonly used protocols** | Participants are instructed to stand on one leg (often with arms crossed over body) and timed until they lose their balance (i.e., stance foot shifts or lifted foot is replaced on the floor)  Common variations in protocol^(38,39)^:  *Maximum time:* Usually 30s, but could be 5s, 15s, 45s, 60s or > 60s  *Floor surface:* hard or foam  *No. of trials:* ranges from 1 to 5 (using either maximum or average of all trials)  *Eyes:* open or closed (or two trials performed with eyes open in first trial and eyes closed in second trial)  *Shoes:* on or off (sometimes both)  *Leg used:* preferred/self-selected, dominant or non-dominant, or both sides | Participants are timed performing a sequence of different standing positions of increasing difficulty:   1. side by side (also called Romberg)   participant stands with feet placed together side by side   1. semi-tandem   participant stands with the side of the  heel of one foot touching the big toe of  the other foot   1. tandem stand (Sharpened Romberg)   participant stands with the heel of one foot in front of and touching the toes of the other foot  Only those participants successfully able to perform one stand for 10s are asked to perform the subsequent stand in the sequence.  Common variations in protocol^(40)^:  *Maximum time:* Each of the 3 stands usually performed for up to 10s whereby maximum test time is 30s but this varies  *Floor surface:* hard or foam  *Eyes:* open or closed  *Shoes:* on or off | Participants are seated and timed placing and/or removing a set number of pegs from a set of holes in a pegboard.  Common variations in protocol:  *Number of pegs and holes:* Number varies between studies, 9 or 25 holes and pegs often used  *Hand used:* preferred/self-selected, dominant or non-dominant, or both  *Instructions:* Remove or place pegs or do both  *Order of peg placement:* Pegs to be placed in a specified order or placed in order of participant’s choosing  *Assessment*: Time to complete task or number of pegs inserted within a set time, with or without penalties |
| **Protocol recommended by NIH toolbox** | Participant assumes and maintains up to 5 poses each for 50 seconds. The sequence of poses is: eyes open on a solid surface, eyes closed on solid surface, eyes open on foam surface, eyes closed on foam surface, eyes open in tandem stance. Postural sway is recorded for each pose using an accelerometer that the participant wears at waist level. | See OLS stands (NIH toolbox combines elements of an OLS with tandem stands) | Time taken for participant to accurately place and remove 9 plastic pegs into a plastic pegboard recorded. One practice and one timed trial performed for each hand. |
| **Reports of validity and reliability** | See references (including review by Yim-Chiplis and Talbot)^(41-43)^ | See references (including review by Yim-Chiplis and Talbot)^(41,43,44)^ | See references^(45-50)^ |
| **Equipment required** | Stopwatch | Stopwatch | Pegboard (molded dish with set number of holes and same number of pegs); stopwatch |
| **Exclusion criteria** | Unable to stand or walk unaided | As for OLS stands | Severe cognitive or comprehension deficits that prevent participants from following verbal commands; Hand injuries |
| **Other measurement considerations** | Ceiling effects at younger ages. Floor effects at older ages. | As for OLS stands | - |
| **Underlying functions** | BALANCE  Muscle strength, core stability, vestibular function, central nervous system function, vision, somatosensory system^(41,51)^ | As for OLS stands | DEXTERITY  Fine manual dexterity; information processing; coordination |
| **Other relevant information** | Wide variability in performance, especially in older populations where even many healthy adults are unable to stand on one leg for even a few seconds. Greater reliability and sensitivity to ageing in tests with eyes open^(39)^.  Reference values reported^(38,42)^.  Several different scales for the measurement of balance have been proposed and widely used^(52,53)^. | In very old populations these stands may be more appropriate than the OLS. | Reference values reported^(48,50,54)^  Influence of handedness on test performance^(55)^.  A range of different tests of dexterity are used in studies^(46)^. |

**References**

(1) Bohannon RW (2006) Reference values for the timed up and go test: A descriptive meta-analysis. *J Geriatr Phys Ther* **29,** 64-68.

(2) Fritz S, Lusardi M (2009) White paper: "walking speed: the sixth vital sign". *J Geriatr Phys Ther* **32,** 46-49.

(3) Fritz SL, Peters DM, Greene JV (2012) Measuring walking speed: Clinical feasibility and reliability. *Top Geriatr Rehabil* **28,** 91-96.

(4) Steffen TM, Hacker TA, Mollinger L (2002) Age- and gender-related test performance in community-dwelling elderly people: Six-Minute Walk Test, Berg Balance Scale, Timed Up & Go Test, and gait speeds. *Phys Ther* **82,** 128-137.

(5) Peters DM, Fritz SL, Krotish DE (2013) Assessing the reliability and validity of a shorter walk test compared with the 10-meter walk test for measurements of gait speed in healthy, older adults. *J Geriatr Phys Ther* 36, 24-30.

(6) Podsiadlo D, Richardson S (1991) The Timed Up and Go - A test of basic functional mobility for frail elderly persons. *J Am Geriatr Soc* **39,** 142-148.

(7) Rockwood K, Awalt E, Carver D *et al*. (2000) Feasibility and measurement properties of the functional reach and the timed up and go tests in the Canadian Study of Health and Aging. J Gerontol A Biol Sci Med Sci **55,** M70-M73.

(8) Salarian A, Horak FB, Zampieri C *et al*. (2010) iTUG, a sensitive and reliable measure of mobility. *IEEE Trans Neural Syst Rehabil Eng* **18,** 303-310.

(9) Lusardi MM (2012) Is walking speed a vital sign? Absolutely. *Top Geriatr Rehabil* **28,** 67-76.

(10) Bohannon RW, Andrews AW, Thomas MW (1996) Walking speed: Reference values and correlates for older adults. *J Orthop Sports Phys Ther* **24,** 86-90.

(11) Bohannon RW (1997) Comfortable and maximum walking speed of adults aged 20-79 years: Reference values and determinants. *Age Ageing* **26,** 15-19.

(12) Graham JE, Ostir GV, Kuo YF *et al*. (2008) Relationship between test methodology and mean velocity in timed walk tests: A review. *Arch Phys Med Rehabil* **89,** 865-872.

(13) Weiss CO, Seplaki CL, Wolff JL *et al*. (2008) Self-selected walking speed was consistent when recorded while using a cane. *J Clin Epidemiol* **61,** 622-627.

(14) Pondal M, del Ser T (2008) Normative data and determinants for the timed "up and go" test in a population-based sample of elderly individuals without gait disturbance. *J Geriatr Phys Ther* **31,** 57-63.

(15) Viccaro LJ, Perera S, Studenski SA (2011) Is timed up and go better than gait speed in predicting health, function, and falls in older adults? *J Am Geriatr Soc* **59,** 887-892.

(16) Kalula SZ, Swingler GH, Sayer AA *et al*. (2010) Does chair type influence outcome in the timed "Up and Go" test in older persons? *J Nutr Health Aging* **14,** 319-323.

(17) Roberts HC, Denison HJ, Martin HJ *et al*. (2011) A review of the measurement of grip strength in clinical and epidemiological studies: towards a standardised approach. *Age Ageing* **40,** 423-429.

(18) Bohannon RW (2011) Test-retest reliability of the five-repetition sit-to-stand test: a systematic review of the literature involving adults. *J Strength Cond Res* **25,** 3205-3207.

(19) Abizanda P, Navarro JL, Garcia-Tomas MI *et al*. (2012) Validity and usefulness of hand-held dynamometry for measuring muscle strength in community-dwelling older persons. *Arch Gerontol Geriatr* **54,** 21-27.

(20) Bohannon RW, Bubela DJ, Magasi SR *et al*. (2011) Relative reliability of three objective tests of limb muscle strength. *Isokinet Exerc Sci* **19,** 77-81.

(21) Watanabe T, Owashi K, Kanauchi Y *et al*. (2005) The short-term reliability of grip strength measurement and the effects of posture and grip span. *J Hand Surg Am* **30,** 603-609.

(22) Bohannon RW, Schaubert KL (2005) Test-retest reliability of grip-strength measures obtained over a 12-week interval from community-dwelling elders. *J Hand Ther* **18,** 426-427.

(23) Wagert PV, Gustafson Y, Lundin-Olsson L (2009) Large variations in walking, standing up from a chair, and balance in women and men over 85 years: an observational study. *Aust J Physiother* **55,** 39-45.

(24) Bohannon RW, Bubela DJ, Magasi SR *et al*. (2010) Sit-to-stand test: Performance and determinants across the age-span. *Isokinet Exerc Sci* **18,** 235-240.

(25) Lord SR, Murray SM, Chapman K *et al*. (2002) Sit-to-stand performance depends on sensation, speed, balance, and psychological status in addition to strength in older people. *J Gerontol A Biol Sci Med Sci* **57,** M539-M543.

(26) Schenkman M, Hughes MA, Samsa G *et al*. (1996) The relative importance of strength and balance in chair rise by functionally impaired older individuals. *J Am Geriatr Soc* **44,** 1441-1446.

(27) Csuka M, McCarty DJ (1985) Simple method for measurement of lower extremity muscle strength. *Am J Med* **78,** 77-81.

(28) Bohannon RW (2006) Reference values for the five-repetition sit-to-stand test: A descriptive meta-analysis of data from elders. *Percept Mot Skills* **103,** 215-222.

(29) Bohannon RW (2008) Is it legitimate to characterize muscle strength using a limited number of measures? *J Strength Cond Res* **22,** 166-173.

(30) Bohannon RW (2012) Are hand-grip and knee extension strength reflective of a common construct? *Percept Mot Skills* **114,** 514-518.

(31) Bohannon RW, Magasi SR, Bubela DJ *et al*. (2012) Grip and knee extension muscle strength reflect a common construct among adults. *Muscle Nerve* **46,** 555-558.

(32) Boadella JM, Kuijer PP, Sluiter JK *et al*. (2005) Effect of self-selected handgrip position on maximal handgrip strength. *Arch Phys Med Rehabil* **86,** 328-331.

(33) Guerra RS, Amaral TF (2009) Comparison of hand dynamometers in elderly people. *J Nutr Health Aging* **13,** 907-912.

(34) Mathiowetz V (2002) Comparison of Rolyan and Jamar dynamometers for measuring grip strength. *Occup Ther Int* **9,** 201-209.

(35) Massy-Westropp N, Rankin W, Ahern M *et al*. (2004) Measuring grip strength in normal adults: reference ranges and a comparison of electronic and hydraulic instruments. *J Hand Surg Am* **29,** 514-519.

(36) Massy-Westropp NM, Gill TK, Taylor AW *et al*. (2011) Hand grip strength: age and gender stratified normative data in a population-based study. *BMC Res Notes* **4,** 127.

(37) Schlussel MM, dos Anjos LA, de Vasconcellos MTL *et al*. (2008) Reference values of handgrip dynamometry of healthy adults: A population-based study. *Clin Nutr* **27,** 601-607.

(38) Bohannon RW (2006) Single limb stance times - A descriptive meta-analysis of data from individuals at least 60 years of age. *Top Geriatr Rehabil* **22,** 70-77.

(39) Michikawa T, Nishiwaki Y, Takebayashi T *et al*. (2009) One-leg standing test for elderly populations. *J Orthop Sci* **14,** 675-685.

(40) Ceria-Ulep CD, Grove J, Chen R *et al*. (2010) Physical aspects of healthy aging: assessments of three measures of balance for studies in middle-aged and older adults. *Curr Gerontol Geriatr Res* **2010,** 849761.

(41) Yim-Chiplis PK, Talbot LA (2000) Defining and measuring balance in adults. *Biol Res Nurs* **1,** 321-331.

(42) Springer BA, Marin R, Cyhan T *et al*. (2007) Normative values for the unipedal stance test with eyes open and closed. *J Geriatr Phys Ther* **30,** 8-15.

(43) Curb JD, Ceria-Ulep CD, Rodriguez BL *et al*. (2006) Performance-based measures of physical function for high-function populations. *J Am Geriatr Soc* **54,** 737-742.

(44) Butler AA, Menant JC, Tiedemann AC *et al*. (2009) Age and gender differences in seven tests of functional mobility. *J Neuroeng Rehabil* **30,** 6.

(45) Wang YC, Magasi SR, Bohannon RW *et al*. (2011) Assessing dexterity function: a comparison of two alternatives for the NIH Toolbox. *J Hand Ther* **24,** 313-20.

(46) Yancosek KE, Howell D (2009) A narrative review of dexterity assessments. *J Hand Ther* **22,** 258-269.

(47) Tiffin J, Asher EJ (1948) The Purdue pegboard; norms and studies of reliability and validity. *J Appl Psychol* **32,** 234-247.

(48) Desrosiers J, Hebert R, Bravo G *et al*. (1995) The Purdue Pegboard Test: normative data for people aged 60 and over. *Disabil Rehabil* **17,** 217-224.

(49) Buddenberg LA, Davis C (2000) Test-retest reliability of the Purdue Pegboard Test. *Am J Occup Ther* **54,** 555-558.

(50) Oxford GK, Vogel KA, Le V *et al*. (2003) Adult norms for a commercially available Nine Hole Peg Test for finger dexterity. *Am J Occup Ther* **57,** 570-573.

(51) Jancová J (2008) Measuring the balance control system-review. *Acta Medica (Hradec Kralove)* **51,** 129-137.

(52) Panella L, Tinelli C, Buizza A *et al*. (2008) Towards objective evaluation of balance in the elderly: validity and reliability of a measurement instrument applied to the Tinetti test. International *J Rehabil Res* **31,** 65-72.

(53) Whitney SL, Poole JL, Cass SP (1998) A review of balance instruments for older adults. *Am J Occup Ther* **52,** 666-671.

(54) Ruff RM, Parker SB (1993) Gender- and age-specific changes in motor speed and eye-hand coordination in adults: normative values for the Finger Tapping and Grooved Pegboard Tests. *Percept Mot Skills* **76,** 1219-1230.

(55) Bryden PJ, Roy EA (2005) A new method of administering the Grooved Pegboard Test: performance as a function of handedness and sex. *Brain Cogn* **58,** 258-268.
